# A protocol to examine vision and gait in Parkinson’s disease: impact of cognition and response to visual cues

**DOI:** 10.12688/f1000research.7320.2

**Published:** 2016-03-24

**Authors:** Samuel Stuart, Brook Galna, Sue Lord, Lynn Rochester

**Affiliations:** 1 Institute of Neuroscience/ Newcastle University Institute for Ageing, Clinical Ageing Research Unit, Newcastle University, Newcastle, NE1 7RU, UK

**Keywords:** Parkinson’s disease, eye-tracking, vision, cognition, visual sampling, gait, eye movements

## Abstract

**Background**

Cognitive and visual impairments are common in Parkinson’s disease (PD) and contribute to gait deficit and falls. To date, cognition and vision in gait in PD have been assessed separately. Impact of both functions (which we term ‘visuo-cognition’) on gait however is likely interactive and can be tested using visual sampling (specifically saccadic eye movements) to provide an online behavioural measure of performance. Although experiments using static paradigms show saccadic impairment in PD, few studies have quantified visual sampling during dynamic motor tasks such as gait.

This article describes a protocol developed for testing visuo-cognition during gait in order to examine the: 1) independent roles of cognition and vision in gait in PD, 2) interaction between both functions, and 3) role of visuo-cognition in gait in PD.

**Methods **

Two groups of older adults (≥50 years old) were recruited; non-demented people with PD (n=60) and age-matched controls (n=40). Participants attended one session and a sub-group (n=25) attended two further sessions in order to establish mobile eye-tracker reliability. Participants walked in a gait laboratory under different attentional (single and dual task), environmental (walk straight, through a door and turning), and cueing (no visual cues and visual cues) conditions. Visual sampling was recorded using synchronised mobile eye-tracker and electrooculography systems, and gait was measured using 3D motion analysis.

**Discussion **

This exploratory study examined visuo-cognitive processes and their impact on gait in PD. Improved understanding of the influence of cognitive and visual functions on visual sampling during gait and gait in PD will assist in development of interventions to improve gait and reduce falls risk. This study will also help establish robust mobile eye-tracking methods in older adults and people with PD.

## Introduction

Parkinson’s disease (PD) is a common neurodegenerative disease ^1^ characterised by the death and dysfunction of dopaminergic neurons in the substantia nigra ^2^. PD causes progressive motor symptoms such as problems with gait ^3^ and non-motor symptoms such as visual and cognitive impairment ^1^. Cognitive impairment is common in PD with reports of dementia ranging up to ~80% ^4^, and may occur early in the disease process ^5^. Visual dysfunction is also common in people with PD, with up to 78% of people with PD reporting at least one visual problem ^6^. Gait impairment in PD is complex, involving multi-system dysfunction and has been widely related to cognitive, and to a lesser extent visual deficits. A more robust understanding of these complex processes and their interactions will inform underlying mechanisms of gait impairment in PD, which may provide insight for future therapeutic intervention. Interventions, such as visual cues (prompts; transverse tape lines to step over) are currently used to ameliorate features of gait disturbance in PD resistant to dopaminergic medication, such as festination, hesitation and freezing of gait ^7, 8^. However, visual cue response is selective ^9^ and the mechanisms that contribute to the response are unclear.

To date, associative (correlational) and online manipulation (via dual tasks and environmental changes) studies have investigated the independent contribution of cognition and vision in gait in PD. However, cognitive and visual functions likely interact and have a combined - impact on gait in PD. Recent technological progress has enabled the monitoring of online visuo-cognition through behavioural outcomes such as visual sampling which reflects both visual ^10, 11^ and cognitive ^12– 14^ processes. Visual sampling is the combination of saccadic fast eye-movements and fixations (pauses between saccades on areas of interest) made during real-world activities ^15^. However, research is compromised by several technological limitations which need to be addressed to ensure robust data collection and analysis. For example, there is currently no ‘gold standard’ visual sampling measurement device or outcome measure and there is also a lack of device accuracy or reliability reporting in all previous studies ^15^.

Visual sampling (specifically saccades) allow orientation to the visual environment bringing areas of interest into high visual acuity (foveation or focus) ^16^. Saccades are impaired in PD and exhibit reduced speed, amplitudes and latencies ^17– 22^. Impaired saccadic eye movements, with reduced latencies and increased error rates have also been reported in PD dementia and dementia with Lewy Bodies, further implicating central neuro-degeneration as a determinant of ocular motor function ^23, 24^. However, the specific contribution of cognitive and/or visual functions to visual sampling during gait in PD and how this impacts gait deficit is currently poorly understood.

Much of the previous saccadic activity research is limited due to the almost exclusive use of static testing protocols (e.g. computerised tasks in sitting) ^18, 25^, which may not be applicable to real-world situations. A recent review of dynamic motor tasks (e.g. gait, obstacle crossing, turning etc.) in PD and older adults ^15^, demonstrated that visual sampling is task dependent and relates to specific goals ^26^. For example: during locomotion over even terrain, saccades may not be required. Over uneven (complex) terrain or during turning saccadic frequency, amplitude and fixations increase ^27– 30^. However many previous visual sampling protocols during dynamic task studies use small cohorts and often do not assess cognitive or visual functions ^15^, which limits interpretation and conclusions regarding underlying mechanisms. Visual sampling during gait therefore has not been fully investigated and further research is required to understand this important feature of gait control. Improved understanding will assist with interventions to improve gait performance in PD.

## Aims

The aims of this study are to better understand: 1) the independent roles of cognition and vision in gait in PD, 2) the interaction between both functions (termed visuo-cognition), and 3) the role of visuo-cognition in gait in PD.

Secondary aims were to:

1. Investigate accuracy and reliability of mobile eye-tracking during gait in people with PD and older adults

## Methods/Design

### Study design

We used a repeated-measures observational design of visual sampling during gait. We also embedded accuracy and reliability testing of a mobile eye-tracker within the study. It involved 100 older adult participants who were separated into two groups (people with PD and older adult controls).

### Participants and setting

Two groups of participants were recruited: i) People with idiopathic PD (PD) (n=60); and ii) Age-matched older adults (controls) (n=40). Inclusion criteria and exclusion criteria are highlighted in Table 1. Vision-specific criteria (identified through medical notes) were included due to the impact of certain conditions on eye-tracking capabilities. The setting for the study was the gait laboratory at the Clinical Ageing Research Unit (CARU), Campus for Ageing and Vitality, Newcastle University, United Kingdom.

**Table 1.** Inclusion and Exclusion Criteria.

Inclusion Criteria	Exclusion Criteria
**Common to all groups** • Aged ≥50 years • Able to walk unaided • Adequate hearing (as evaluated by the whisper test; stand 2m behind participant and whisper a 2 syllable word, participant repeats word) and vision capabilities (as measured using a Snellen chart – 6/18–6/12). • Stable medication for the past 1 month and anticipated over a period of 6 months **Group Specific Criteria** **Participants with PD:** • Diagnosis of idiopathic PD, as defined by the UK Brain Bank criteria ^31^ • Hoehn and Yahr stage I–III ^32^ • Stable medication for past 1 month and anticipated over next 6 months or stable Deep Brain Stimulation for at least one month and expected following 6 months • Score ≥21/30 on Montreal cognitive assessment (MoCA) which is used to classify non-demented PD (PD dementia is <21/30) ^33– 35^ • Free from any neurological disorders that may have caused cognitive impairment • No restriction was made for medication usage and participants on stable doses of medication or treatment were permitted.	**Common to all groups** • Psychiatric co-morbidity (e.g., major depressive disorder as determined by geriatric depression scale (GDS-15); >10/15 ^36^) • Clinical diagnosis of dementia or other severe cognitive impairment (PD = MoCA <21/30, Controls = MoCA <26/30 ^37^) • History of stroke, traumatic brain injury or other neurological disorders (other than PD, for that group) • Acute lower back or lower extremity pain, peripheral neuropathy, rheumatic and orthopaedic diseases • Unstable medical condition including cardio-vascular instability in the past 6 months • Unable to comply with the testing protocol or currently participating in another interfering research project • Interfering therapy **Vision Specific Criteria** • Any pupillary diameter disorder; such as significantly non-round pupils, Adies pupil (tonic or dilated pupil), Argyll- Robertson pupil (absence of light reaction), unilateral small pupil • Neuromotility disorders, such as Nystagmus or other ocular oscillations • Significant left eye disorders (i.e. squint, twitching, Ptosis [drooping eyelids]) • Known significant visual field deficits; such as hemianopia • Optic nerve disease • Optic disc elevation • Optic disc swelling; such as Papilledema or Papillitis

### Recruitment

People with PD were identified through the Movement Disorders Clinic at the Clinics for Research and Service in Themed Assessments (CRESTA) in Newcastle upon-Tyne. Research personnel were available at clinics as required to invite participants to consider the study. If sufficiently interested, participants were given a Participant Information Sheet (PIS) and letter concerning the study. The invitation was followed up by a telephone call during the week to assess willingness to participate. If willing, a mutually convenient time for assessment was organised and the invitation to attend was extended to a carer or spouse.

The older adult control group was recruited via advertisement using posters placed within neurology and geriatric departments. The advertisement was sent via the university email system to staff and students at Newcastle University. Recipients were asked to pass on the poster to potential interested parties (i.e. family or friends). Participants received reimbursement of travel expenses for their own vehicle or for public transport, if this is preferred.

## Measures and procedures

### Global cognitive assessment

Global cognition was assessed using the Montreal cognitive assessment (MoCA) and Addenbrookes cognitive examination (ACE-R) ^37^. The MoCA was performed during screening to exclude control participants with cognitive impairment (MoCA <26) and PD participants with dementia (MoCA <21) ^5^ ( Table 1). The MoCA is a valid and standardized neuropsychological test for rapid screening of global cognitive dysfunction ^37^, and assesses several different cognitive domains (attention and concentration, executive functions, memory, language, visuo-constructional skills, conceptual thinking, calculations, and orientation). ACE-R has also been shown to be valuable in differential diagnosis of PD when compared to the mini-mental state examination (MMSE) ^38^. Similar to the MoCA, the ACE-R involves testing multiple cognitive domains, such as; attention, orientation, memory, fluency, language and visuospatial abilities.

### Specific Cognitive Domain Assessment

***Attention.*** Attention was measured via the Cognitive Drug Research (CDR) battery (United Biosource Corporation, UK). This provides specific measures of attention, including Power of attention which is the sum of Simple reaction time, Digit vigilance and Choice reaction time ^39^. The attention CDR is a valid test of attention and has been used in a number of studies involving both PD and cognitively impaired individuals ^40^. The attention CDR involves a series of computerised tests, which the participants respond to by pressing one of two buttons (YES or NO buttons).

***Executive function.*** Clock drawing (specifically Royall’s CLOX 1) ^41^ was used as a measure of executive function (i.e. planning). Clock drawing assessment is a measure of cognitive impairment, which is an internally consistent measure that is easy to administer and has good reliability. Participants were required to plan and draw a clock from memory with the numbers and arrows pointed at a particular time, which is then marked out of 15 for certain criteria (e.g. hour hand shorter than the minute hand = one point).

***Working Memory.*** Working memory was assessed using the maximal Wechsler forward digit span ^42^, performed while seated. The forward digit span is reported as a simple span test, which measures storage and manipulation of information by working memory ^43^.

The forward digit span consists initially of two numbers being played over loud speaker at a rate of 1 per second for the participant to recall, and continues to a maximum of nine numbers ^43^. Three trials per span length were conducted and the test continued until a participant fails two out of three trials. The maximal length of the digit span was determined, defined as the most numbers a participant could remember two out of three times without error.

### Visuo-spatial assessment

Clock copying (specifically Royall’s CLOX 2) ^41^ measured visuo-spatial ability (i.e. ability to identify the spatial relationship of objects). Clock copying is considered a valid measure of visuo-spatial ability linked with right parietal pathology ^41, 44^. For CLOX 2 the researcher draws a clock and the participant must then copy the clock drawn, similar to the cube copying in the MoCA.

Benton’s Judgement of Line Orientation (JLO) test was also used as a measure of visuo-spatial ability. The JLO test has been shown to be a valid and reliable measure of visuo-spatial abilities ^45^. The JLO test involves a participant viewing a set of numbered lines and then being shown two lines of the same orientation. They then have to name the numbers that the shown lines correspond to.

Specific sections of the visual object and space perception (VOSP) battery was used for more specific visuo-spatial assessment, such as; incomplete letters (visual object perception), dot counting and position discrimination (both spatial perception). The VOSP has been shown to be a valid measure of visuo-spatial abilities ^46^ and consists of a screening test to establish requisite sensory acuity and specific clinical tests ^47^. The VOSP test has been used before in older adults and neurological disorder studies ^48– 50^.

### Visual Function Assessment

Visual function assessment included measurement of visual acuity (VA) and contrast sensitivity (CS) using basic eye-charts.

***Visual acuity (VA).*** VA was measured binocularly using a standard LogMAR chart ^51^. Participants were seated at a distance of 4m from the chart. Participants were instructed to read aloud down the chart starting from the top left. All correct answers are recorded on a pre-set score sheet. The test is terminated if the participant makes two consecutive errors ^52^. Assessment was done for each eye and binocularly.

***Contrast sensitivity (CS).*** CS was measured using the Mars CS sheets (Mars letter CS chart, Mars Percetrix™, New York, USA) placed on an adjustable holder ^53^. The sheet consists of 48 Latin letters of uniform height; the contrast from the white background decreases with subsequent letters. Room illumination was adjusted so that average CS sheet luminance was between 80 and 120cd/m² (measured via a luminance meter). Assessment was done for each eye and binocularly with the average distance from the participants eyes being 50cm. Participants read aloud down the sheet starting at the top left. Errors were recorded on the pre-set score sheet and testing was terminated after two consecutive errors.

### Parkinson’s disease-specific assessment

***The Unified Parkinson's disease Rating Scale (UPDRS).*** The Unified Parkinson's Disease Rating Scale ^54^ (Movement Disorder Society revised version) was used to assess motor and non-motor features of PD and disease severity. The UPDRS was scored from a total of 195 points; higher scores reflect worsening disability.

***Hoehn & Yahr (H & Y).*** The Hoehn and Yahr rating scale ^55^ is a widely used clinical rating scale, which defines broad categories of motor function in PD. Only PD participants with mild to moderately severe motor function (H&Y stages I–III) were included.

***The FOG questionnaire (FOGQ).*** Freezing of gait (FOG) was evaluated using the FOG questionnaire ^56, 57^. This is a ten-item questionnaire intended to classify FOG. The questionnaire has three parts; distinction of freezers from non-freezers, freezing severity, frequency and duration and impact of freezing on daily life.

### Assessments common to both groups

***The Geriatric Depression Scale (GDS-15) short form.*** The geriatric depression scale (GDS-15) short form ^54, 55^ was used to evaluate participant depression. The GDS-15 was created in 1986 by Sheikh and Yesavage and involves 15 questions about the mood of participants ^56^. The GDS-15 classifies depression via the following scores; 0 to 4 indicates a normal range, 5 to 9 indicates mild depression, and 10 to 15 indicates moderate to severe depression ^57^.

***Falls Efficacy Scale – International version (FES-I).*** Fear of falling was measured using the falls efficacy scale – international version (FES-I). This is a short validated measure of fear of falling in older adults, which assesses basic and demanding activities (both physical and social) ^58^. It consists of 16 scenarios (e.g. cleaning the house) and participants must rate their fear of falling on a scale from 1 (Not at all concerned) to 4 (Very concerned).

## Measurement of visual sampling during gait

**Figure 1.**
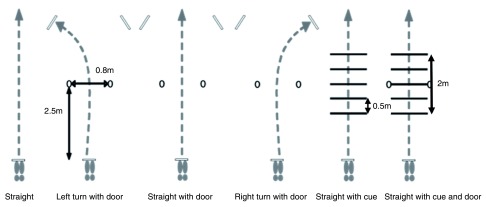
Walking conditions.

Participants walked under different *environmental* ( Figure 1) and *attentional* conditions in order to assess the impact of more complex (visual) environments and (cognitive) tasks.

Environmental conditions included; walking straight, walking straight through a doorway and turning while walking through a doorway (see Figure 1). The visual sampling during gait testing was also repeated with a visual cue in place for the straight walks. The visual cue consisted of transverse black tape lines on a white floor placed 50cm apart (approx. a ‘normal’ step length) as depicted in Figure 1, which participants were asked to step over as they complete the walk. A visual cue was used as they are known to help ameliorate gait impairments in PD ^61^, which may be due to the increased task-related visual information ^62^ or greater attention being allocated to gait ^61^.

Attentional conditions included; single task (i.e. just walking) and dual task (i.e. repeating numbers while walking based on a maximal forward digit span obtained in sitting). A dual task was used as a representative of real-world walking, in which carrying out several tasks at once is common (i.e. walking and talking) ^60^.

Both groups (PD and controls) performed the same walking conditions ( Figure 1); with repeat measures (three trials for each condition) taken for an average to be created.

### Equipment

Visual sampling (the combination of saccades and fixations) was assessed with a Dikablis (Ergoneers, Germany) head-mounted infra-red eye tracking system, synchronised with a 3D motion capture system (Vicon, Oxford, UK) and an electrooculography (EOG) system (Zerowire, Aurion, Italy), to allow for simultaneous and comprehensive recording and analysis of gait and eye movement data. Dikablis calibration was performed while standing using the manufacturer 4-point procedure for each participant prior to data collection. Similar to our previous research ^29^, EOG was also calibrated prior to data collection via asking participants to blink for 30 secs and move their eyes horizontally between set-distance visual targets (5°, 10° and 15°) for 30 secs in time with an auditory cue (a metronome beat) while seated.

The Dikablis eye-tracker recorded eye movement using an infra-red camera ^63– 65^, this data was combined with EOG data which involves two small electrodes being applied bi-temporally on the forehead of the participant. Importantly, the Dikablis has an adequate sampling frequency (50Hz) to detect saccades during gait ^66, 67^ and EOG has a high sampling frequency (1000Hz) which allows accurate acquisition of specific visual sampling characteristics such as velocity, acceleration, distance etc. ^15^. The Dikablis device includes two aspects; a head unit and a transmitter bag. Both the head unit (approx. the same size as a pair of glasses) and the bag (approx. 1kg) are lightweight. The head unit was taped, with a small amount of double sided tape, to the forehead of the participants to prevent error due to slippage. Eye movement data from the Dikablis was collected at 50Hz and from the EOG system at 1000Hz; this was saved onto a computer to be analysed using proprietary software ^66^.

**Figure 2.**
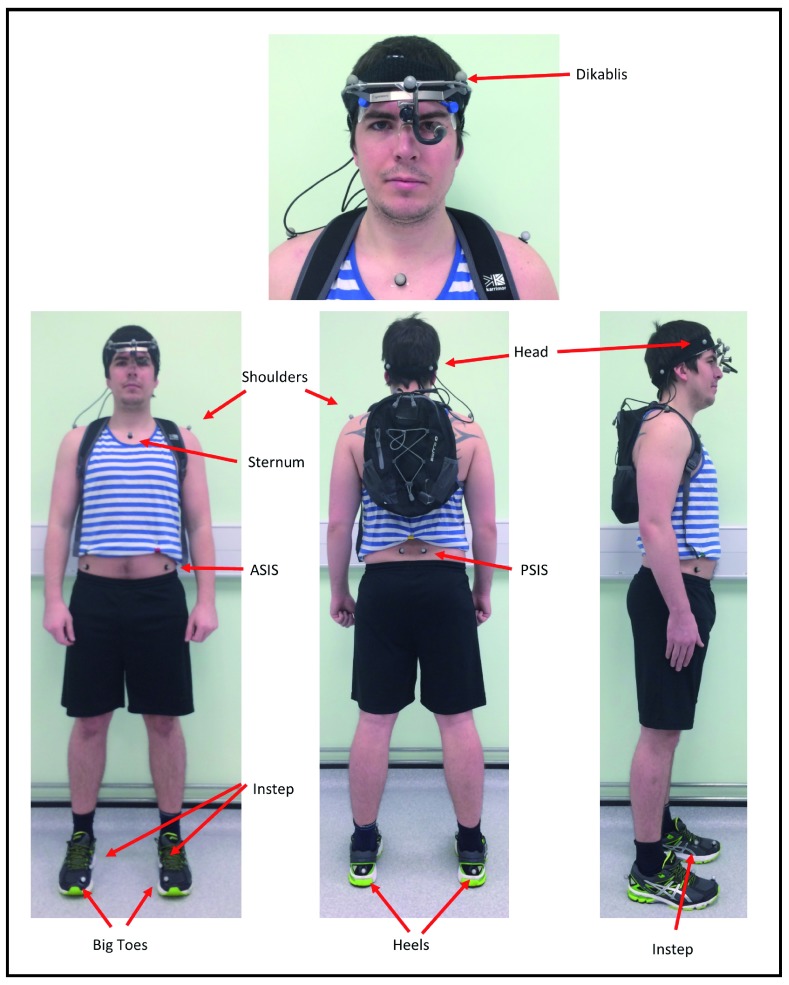
Reflective marker body placement locations.

Video recording and the Vicon 3D motion capture system recorded participants movement during walking using a camcorder and infra-red sensors attached to the skin of the participants at specific locations ( Figure 2; 2× shoulders, 1× sternum, 2× anterior superior iliac spine (ASIS), 2× posterior superior iliac spine (PSIS), 2× big toe, 2× instep, 2× heel and 4× head) using a small amount of double sided tape. Participants were required to bring their own shorts and a vest to wear in order for the markers to be placed onto the appropriate body locations. Vicon 3D motion analysis is a valid and reliable method of assessing the spatiotemporal parameters of gait in older adults and in people with PD ^68^.

### Accuracy and reliability testing of visual sampling

Mobile infra-red eye-tracking and EOG have been shown to be a valid and reliable method for assessing saccadic activity in younger adults ^69^, and both have previously been used in older adults and in people with PD ^29, 70– 73^. We were interested in the accuracy and test-retest reliability of mobile eye-tracking in people with PD and older adult controls to ensure the robustness of data interpretation. Therefore, a subgroup (PD and control; up to n=25) were asked to return approx. one week later for a second and third visit for accuracy and test re-test reliability testing ( Table 2). The Dikablis eye-tracker recorded eye movement and was used in the same manner as the previous study ^63– 65^, combined with video recording of individuals body movement and a tri-axial accelerometer (Axivitiy, AX3, York, UK) recording head movement.

**Table 2.** Study protocol overview.

Participants (n = 100)	Session 1 (up to 150min)	Session 2 (up to 60min)	Session 3 (up to 60min)
1. Older adult controls (n = 40) 2. Parkinson’s disease (n = 60)	Applicable to all participants (n=100) • Initial screening, cognitive and visual function assessments (45–60min) • Informed consent • Demographic and diagnostic evaluation **Global cognitive assessments:** • MoCA • ACE-R **Specific cognitive domain** **assessment:** • JLO • CLOX 1 and 2 • VOSP battery • Attention CDR battery **Visual function assessments:** • Visual acuity • Contrast sensitivity **PD-Specific assessments:** • UPDRS • Hoehn and Yahr • FOG-questionnaire **Common assessments:** • GDS-15 • FES-I Visual sampling during gait testing in gait laboratory (60–90min)	Applicable; for a subgroup of PD and control participants (n=25) Approx. 1 week after session 1 1 ^st^ Reliability testing (45–60min) • Repeat visual sampling during gait testing in gait laboratory (single task, without a visual cue) • Sit, stand and walk on a treadmill while making eye- movements to set distance targets (5°, 10° and 15°)	Applicable; for a subgroup of PD and control participants (n=25) Approx. 1 week after session 2 2 ^nd^ Reliability testing (45–60min) • Sit, stand and walk on a treadmill while making eye- movements to set distance targets (5°, 10° and 15°)

In the second session the sub-group of participants were asked to repeat the walking tasks from session 1 (single task, without a visual cue) to provide visual sampling during gait reliability data. Accuracy of visual sampling measurement was determined by asking participants to sit (with chin rest *in situ*), stand (without moving their head) and walk (free head movement) on a treadmill, while performing several eye movements to visual targets (horizontal and vertical visual angles such as 5°, 10°, 15°) in time with an auditory cue (a metronome). The subgroup was asked to return for a third visit (within approx. 1 week of the second visit) to repeat the accuracy testing (as above) in order to derive test-retest reliability results.

## Primary outcome measure

### Saccade frequency during gait

The primary outcome measure was saccade frequency (number of fast eye movements per second when walking) during gait, which was recorded via the Dikablis mobile eye-tracker and EOG systems.

### Secondary outcome measures

***Visual sampling.*** Secondary visual sampling outcomes included: saccade number, velocity, acceleration, amplitude and duration, as well as fixation number and duration.

***Gait characteristics.*** Gait characteristics were measured via video recording and a Vicon 3D motion capture system for all walking conditions in order to examine associations between cognitive and visual functions and gait, and saccadic frequency and gait ( Figure 1). Spatiotemporal gait characteristics included step velocity, step length, step time, single support time and double support time, which were chosen because they have been selectively associated with cognitive ^74^ and visual functions ^75, 76^ in people with PD and older adults in previous research.

***Safety considerations.*** All measurements were non-invasive and placed the participant at no risk other than those that normally may occur during walking. To prevent excessive fatigue, participants were encouraged to take breaks as needed throughout all study procedures. The hypoallergenic double-sided tape used to fix the infra-red markers and Dikablis head unit onto the skin of the participants did not cause any adverse effects. The amount of tape was small and it has been used on numerous occasions in other research projects at the CARU and no issues have been reported. The bi-temporal EOG electrodes also did not cause any adverse effects. The treadmill used within the accuracy and reliability testing was equipped with a safety harness to avoid any falls-related injuries, as the harness could support the participant and trigger the treadmill to automatically stop in the event of a fall.

***Ethical approval.*** Ethical approval for this project was obtained from the NRES Committee North East -Newcastle and North Tyneside 1 Research Ethics Committee (approved 6 ^th^ June 2013, Reference 13/NE/0128). Written informed consent was obtained for every participant prior to testing. The study began 1 ^st^ July 2013.

***Dissemination.*** Data collection for the study finished in July 2015 and results will be published within peer reviewed scientific journals, open-access publication will be preferred. A public engagement event will also be used to disseminate findings to participants and public. All participants were assigned participant numbers, allowing data to be anonymised and reported confidentially. All results from the study will be uploaded to Clinicaltrails.gov (ID: NCT02610634) once analysed. No contractual agreement limits access to data.

## Statistical analysis

### Sample size

This was an exploratory study and therefore few specific previous examples were available to guide estimates for sample size. We have based the estimate (≥40 participants in each group) on our previous work (PD; n=21) ^29^ and other previous similar studies. Similar studies in this research area ^72, 73, 77– 80^ have used small sample sizes (n=2–26) and reported between-group differences, demonstrating that we will be able to see differences between our sizable PD and control groups. It is a general recommendation to include 30 cases per group to be able to carry out basic statistical tests (e.g. between group comparisons) ^81^. This study will inform future power calculations.

Data analysis will follow a predetermined plan:

### Analysis common to all studies

Statistical analysis will be undertaken using SPSS version 21 (SPPS, Inc. an IBM company). Demographic characteristics and baseline data will be summarized using descriptive statistics, including means, standard deviations, median, minimum, maximum and inter-quartile ranges for continuous or ordinal data and percentages for categorical data. The descriptive statistics will be tabulated and presented graphically for clarity. One-sample Kolmogorov-Smirnov tests will be used to check for normally distributed data. Non-normally distributed continuous distributions will be transformed where appropriate to meet the requirements of parametric tests; otherwise equivalent non-parametric tests will be adopted. Data will also be assessed graphically (such as histograms or scatter plots) for clarity of information. As this is an exploratory study a threshold of *p* < .05 (two-sided) will guide statistical interpretation. A brief summary of participant demographic and clinical outcomes is provided in Table 3.

**Table 3.** Brief summary of demographic and clinical features.

		Control (n=40) Mean (SD)	PD (n=60) Mean (SD)	***p***
Demographic	Age (years)	66.93 (10.86)	67.77 (7.60)	.649
	Sex	17M/23F	38M/22F	**.041** †
	Education (years)	14.80 (3.03)	13.28 (3.61)	.031 *
	Depression scale (GDS-15)	0.70 (0.88)	2.80 (2.77)	.000 *
	Falls efficacy scale (FES-I)	18.98 (4.15)	25.48 (8.99)	.000 *
Clinical	Hoehn and Yahr stage (H&Y)	-	I (21)/II (33)/III (6)	-
	Disease duration (months)	-	75.38 (75.50)	-
	UPDRS part III	-	37.13 (13.84)	-
	FOGQ	-	4.33 (7.21)	-
	LED	-	629.49 (412.82)	-

*independent t-test significance level p<.05, † = X², LED = levodopa equivalent dose, FOGQ = freezing of gait questionnaire and UPDRS III = unified Parkinson’s disease rating scale (motor subsection)

### Further analysis

Study aims will be addressed using the specific analysis provided below:

***1) To examine the independent roles of cognition and vision in gait in PD***

Associations between cognition, visual functions and gait characteristics will initially be made using Pearson correlations, which will be followed by structural equation modelling (SEM) (detailed below).

***2) To examine the interaction between cognitive and visual functions (termed visuo-cognition)***

Visual sampling (saccade frequency) is an online behavioural measure of visuo-cognition due to its known relationship with cognitive and visual functions ^82^. To analyse visual sampling during gait, a series of mixed analysis of variance (ANOVA) will be used with effect of PD (PD and control) as between participant factor and attention (single task, dual task) and environment (Straight walk, Door, Turn) as within group factors. Pearson’s correlations will be used to test the strength and direction of the relationships between clinical, gait and saccade frequency outcomes. Gait characteristics will also be assessed with the same mixed ANOVA method.

To test the effect of visual cueing on visual sampling and gait; a mixed ANOVA will be used with group (PD and control), visual cue (no cue and cue) and attention (single task, dual task). Comparison with and without a visual cue will also be made via the same mixed ANOVA for the various gait characteristics, while controlling for the influence height.

Associations between cognitive and visual functions will be made using Pearson correlations. Cognitive and visual function contribution to visual sampling will be assessed using multiple regression analysis, while controlling for demographic factors (age, motor severity, depression, global cognition). This will be performed in several steps; Step 1: Demographics, Step 2: Cognition (attention, executive function, visuo-spatial ability, working memory), Step 3: Visual functions (visual acuity, contrast sensitivity), and Step 4: Visuo-cognition (combination of all of the variables in the above steps).

***3) To examine the role of visuo-cognition in gait in PD***

SEM will be used to assess an *a priori* hypothesised model of visuo-cognition in gait in PD ^82^. This model will examine the inter-relationships between cognition, visual function, visual sampling (saccade frequency) and gait in PD. SEM is an ideal statistical method for assessing *a priori* hypotheses, as it allows for hypothesised interactions between variables to be represented within the model. SEM combines ANOVA, correlation, path analysis, factor analysis and regression, and provides direct and indirect relationships between variables, which are not provided by regression analysis ^83^. Direct effects are those where a single path connects one variable to another. Indirect effects are those where the effect of one variable on another goes through a third variable (i.e. more than one path connects two variables) ^84^.

SEM analysis will be conducted using current industry recommendations ^85– 89^. Four steps will be undertaken:

1) Four latent variables will be created (i.e. cognition, visual function, visual sampling and gait) using the same observed variables (e.g. visual acuity) as within the multiple regression analysis.
2) Poor latent variable representations will be removed (i.e. observed variables that do not meet a standardised factor loading of ≥0.70 will be removed for each latent variable ^89, 90^).
3) Any observed variable with a standardised factor loading of ≥1.00 will be used in place of the latent variable to avoid overfitting ^89^.
4) Model trimming and effect calculation; non-significant associations (connection arrows/paths) will be removed, and direct and indirect effects calculated (i.e. for indirect effects coefficients for each path will be multiplied ^91^).

### Secondary analysis

***1. Investigate accuracy and reliability of mobile eye-tracking during gait in people with PD and older adults***

To analyse reliability; repeated-measure *t*-tests, Bland and Altman plots, intra-class correlation coefficients (Model 2, 1) and Pearson’s correlations (or non-parametric equivalents) will be used to assess bias, absolute and relative agreement and consistency of saccadic outcomes measured with the Dikablis eye-tracker on two separate occasions a week apart. A similar statistical approach will be used to assess accuracy of the Dikablis system against targets of a known angle (5°, 10° and 15°).

## Discussion

The aims of this study were to provide a greater understanding of the roles that cognition and vision play in gait in PD. Specifically this study provided data regarding the role that visuo-cognition plays in gait in PD, as well as relationships between cognitive and visual functions (termed visuo-cognition). What sets this project apart from other work in this field is that the study is taking into consideration the combined and interactive impact that cognitive and visual function impairments have on gait in PD.

The study protocol was developed in response to recently reviewed evidence and study recommendations for visual sampling during a dynamic motor task ^15^. The protocol focussed not only on cognitive impairments but also visual dysfunction which is commonly reported in PD and until now has not been fully investigated. Little quantitative data has been previously reported regarding visual sampling during real-world tasks (e.g. gait, reaching etc.) in PD and the few previous studies available only involve small cohorts often performing simple static motor tasks (i.e. mouse clicks or button pressing or reaching ^92, 93^).

This study investigated the online visuo-cognitive behavioural measure of visual sampling during a real-world task (i.e. gait), and data analysis will examine interaction between visual sampling, cognitive and visual functions and task performance. The study will determine the influence of cognitive and visual functions on visual sampling during gait and gait characteristics in PD. This will allow us to determine whether gait impairments in PD are influenced by basic visual function (CS and VA) impairment or cognitive impairment (particularly attention) or a combination of these aspects.

Finally, an important feature of this study is that it is expected to provide the first evidence on the accuracy and reliability of using mobile eye-tracking equipment during gait with older adults and people with PD, which will develop the standard of research being conducted in this area and allow for more definitive conclusions.

## Conclusion

This exploratory observational study will assist with understanding the role that cognition and vision play in gait in PD and how combined visuo-cognitive processes influence gait outcomes. In addition, it will provide evidence on the interaction between cognitive and visual functions in PD, as well as how visual sampling during gait is affected by the use of clinical interventions such as visual cues.

## List of Abbreviations

ACE-R: Addenbrookes cognitive examination (revised version)

ANOVA: analysis of variance

CARU: clinical ageing research unit

CDR: Cognitive drug battery

CRESTA: Clinics for Research and Service in Themed Assessments

CS: Contrast sensitivity

EOG: Electro-oculography

FES-I: Falls efficacy scale (international version)

FOG: Freezing of gait

FOGQ: Freezing of gait questionnaire

GDS-15: Geriatric depression scale (short form)

JLO: Judgement of line orientation

MMSE: Mini mental state examination

MoCA: Montreal cognitive assessment

PD: Parkinson’s disease

PIS: Participant information sheet

UPDRS: Unified Parkinson’s disease rating scale (Movement Disorder Society revised version)

VA: Visual acuity

VOSP: Visual object and space perception battery

## References

[1] Armstrong RA: Visual symptoms in Parkinson's disease. *Parkinsons Dis.* 2011;2011: 908306. 10.4061/2011/908306 21687773 PMC3109513

[2] Antal A Terney D Bodis-Wollner I: Parkinson's Disease, Aging and Visual Cognition. *Top Geriatr Rehabil.* 2008;24(2):166–81. 10.1097/01.TGR.0000318903.80066.e7

[3] Geldmacher DS: Visuospatial dysfunction in the neurodegenerative diseases. *Front Biosci.* 2003;8:e428–36. 10.2741/1143 12957879

[4] Verbaan D Marinus J Visser M: Cognitive impairment in Parkinson's disease. *J Neurol Neurosurg Psychiatry.* 2007;78(11):1182–7. 10.1136/jnnp.2006.112367 17442759 PMC2117586

[5] Aarsland D Bronnick K Williams-Gray C: Mild cognitive impairment in Parkinson disease: a multicenter pooled analysis. *Neurology.* 2010;75(12):1062–9. 10.1212/WNL.0b013e3181f39d0e 20855849 PMC2942065

[6] Davidsdottir S Cronin-Golomb A Lee A: Visual and spatial symptoms in Parkinson's disease. *Vision Res.* 2005;45(10):1285–96. 10.1016/j.visres.2004.11.006 15733961

[7] Nieuwboer A Kwakkel G Rochester L: Cueing training in the home improves gait-related mobility in Parkinson's disease: the RESCUE trial. *J Neurol Neurosurg Psychiatry.* 2007;78(2):134–40. 10.1136/jnnp.200X.097923 17229744 PMC2077658

[8] Frazzitta G Maestri R Uccellini D: Rehabilitation treatment of gait in patients with Parkinson's disease with freezing: a comparison between two physical therapy protocols using visual and auditory cues with or without treadmill training. *Mov Disord.* 2009;24(8):1139–43. 10.1002/mds.22491 19370729

[9] Kompoliti K Goetz CG Leurgans S: "On" freezing in Parkinson's disease: resistance to visual cue walking devices. *Mov Disord.* 2000;15(2):309–12. 10.1002/1531-8257(200003)15:2<309::AID-MDS1016>3.0.CO;2-P 10752582

[10] Hernandez TD Levitan CA Banks MS: How does saccade adaptation affect visual perception? *J Vis.* 2008;8(8):3.1–16. 10.1167/8.8.3 18831626 PMC2630579

[11] Bridgeman B Kirch M Sperling A: Segregation of cognitive and motor aspects of visual function using induced motion. *Percept Psychophys.* 1981;29(4):336–42. 10.3758/BF03207342 7279556

[12] Baluch F Itti L: Mechanisms of top-down attention. *Trends Neurosci.* 2011;34(4):210–24. 10.1016/j.tins.2011.02.003 21439656

[13] Shen J Itti L: Top-down influences on visual attention during listening are modulated by observer sex. *Vision Res.* 2012;65:62–76. 10.1016/j.visres.2012.06.001 22728922

[14] Mazer JA: Spatial attention, feature-based attention, and saccades: three sides of one coin? *Biol Psychiatry.* 2011;69(12):1147–52. 10.1016/j.biopsych.2011.03.014 21529782 PMC3572732

[15] Stuart S Alcock L Galna B: The measurement of visual sampling during real-world activity in Parkinson's disease and healthy controls: a structured literature review. *J Neurosci Methods.* 2014;222:175–88. 10.1016/j.jneumeth.2013.11.018 24291711

[16] Mosimann UP Muri RM Burn DJ: Saccadic eye movement changes in Parkinson's disease dementia and dementia with Lewy bodies. *Brain.* 2005;128(Pt 6):1267–76. 10.1093/brain/awh484 15774501

[17] van Stockum S MacAskill M Anderson T: Don't look now or look away: two sources of saccadic disinhibition in Parkinson's disease? *Neuropsychologia.* 2008;46(13):3108–15. 10.1016/j.neuropsychologia.2008.07.002 18674551

[18] van Stockum S MacAskill MR Anderson TJ: Impairment of voluntary saccades and facilitation of reflexive saccades do not co-occur in Parkinson's disease. *J Clin Neurosci.* 2012;19(8):1119–24. 10.1016/j.jocn.2011.10.014 22705130

[19] van Stockum S Macaskill MR Myall D: A perceptual discrimination task abnormally facilitates reflexive saccades in Parkinson's disease. *Eur J Neurosci.* 2011;33(11):2091–100. 10.1111/j.1460-9568.2011.07697.x 21645103

[20] Muilwijk D Verheij S Pel JJ: Changes in Timing and kinematics of goal directed eye-hand movements in early-stage Parkinson's disease. *Transl Neurodegener.* 2013;2(1):1. 10.1186/2047-9158-2-1 23298720 PMC3563471

[21] Ventre-Dominey J Dominey PF Broussolle E: Dissociable processing of temporal structure in repetitive eye-hand movements in Parkinson's disease. *Neuropsychologia.* 2002;40(8):1407–18. 10.1016/S0028-3932(01)00207-X 11931945

[22] Ventre-Dominey J Ford Dominey P Broussolle E: Asymmetric influences of pointing on saccade latency in hemi-Parkinson's disease. *Neuropsychologia.* 2001;39(5):470–7. 10.1016/S0028-3932(00)00133-0 11254929

[23] Archibald NK Clarke MP Mosimann UP: The retina in Parkinson's disease. *Brain.* 2009;132(Pt 5):1128–45. 10.1093/brain/awp068 19336464

[24] Mosimann UP Mather G Wesnes KA: Visual perception in Parkinson disease dementia and dementia with Lewy bodies. *Neurology.* 2004;63(11):2091–6. 10.1212/01.WNL.0000145764.70698.4E 15596755

[25] Bekkering H Neggers SF Walker R: The preparation and execution of saccadic eye and goal-directed hand movements in patients with Parkinson's disease. *Neuropsychologia.* 2001;39(2):173–83. 10.1016/S0028-3932(00)00092-0 11163374

[26] Marigold DS Patla AE: Gaze fixation patterns for negotiating complex ground terrain. *Neuroscience.* 2007;144(1):302–13. 10.1016/j.neuroscience.2006.09.006 17055177

[27] Land MF: Eye movements and the control of actions in everyday life. *Prog Retin Eye Res.* 2006;25(3):296–324. 10.1016/j.preteyeres.2006.01.002 16516530

[28] Patla AE Greig M: Any way you look at it, successful obstacle negotiation needs visually guided on-line foot placement regulation during the approach phase. *Neurosci Lett.* 2006;397(1–2):110–4. 10.1016/j.neulet.2005.12.016 16413969

[29] Galna B Lord S Daud D: Visual sampling during walking in people with Parkinson's disease and the influence of environment and dual-task. *Brain Res.* 2012;1473:35–43. 10.1016/j.brainres.2012.07.017 22824332

[30] Lohnes CA Earhart GM: Saccadic eye movements are related to turning performance in Parkinson disease. *J Parkinsons Dis.* 2011;1(1):109–18. 10.3233/JPD-2011-11019 22216083 PMC3247902

[31] Hughes AJ Daniel SE Kilford L: Accuracy of clinical diagnosis of idiopathic Parkinson's disease: a clinico-pathological study of 100 cases. *J Neurol Neurosurg Psychiatry.* 1992;55(3):181–4. 10.1136/jnnp.55.3.181 1564476 PMC1014720

[32] Hoehn MM Yahr MD: Parkinsonism: onset, progression and mortality. *Neurology.* 1967;17(5):427–42. 10.1212/01.wnl.0000405146.06300.91 6067254

[33] Bernardi MG: Mild Cognitive Impairment is Under-Recognised in Newly Referred Patients with Parkinson’ s Disease: Mini Mental State Examination (MMSE) Versus Montreal Cognitive Assessment (Moca). *J Alzheimer’ s Disease Parkinsonism.* 2012;01 10.4172/scientificreports.280

[34] Smith T Gildeh N Holmes C: The Montreal Cognitive Assessment: validity and utility in a memory clinic setting. *Can J Psychiatry.* 2007;52(5):329–32. 17542384 10.1177/070674370705200508

[35] Nasreddine ZS Phillips NA Bédirian V: The Montreal Cognitive Assessment, MoCA: a brief screening tool for mild cognitive impairment. *J Am Geriatr Soc.* 2005;53(4):695–9. 10.1111/j.1532-5415.2005.53221.x 15817019

[36] Aikman GG Oehlert ME: Geriatric Depression Scale: Long Form Versus Short Form. *Clinical Gerontologist.* 2001;22(3–4):63–70. 10.1300/J018v22n03_07

[37] Dalrymple-Alford JC MacAskill MR Nakas CT: The MoCA: well-suited screen for cognitive impairment in Parkinson disease. *Neurology.* 2010;75(19):1717–25. 10.1212/WNL.0b013e3181fc29c9 21060094

[38] Rittman T Ghosh BC McColgan P: The Addenbrooke's Cognitive Examination for the differential diagnosis and longitudinal assessment of patients with parkinsonian disorders. *J Neurol Neurosurg Psychiatry.* 2013;84(5):544–51. 10.1136/jnnp-2012-303618 23303961 PMC3623037

[39] Allcock LM Rowan EN Steen IN: Impaired attention predicts falling in Parkinson's disease. *Parkinsonism Relat Disord.* 2009;15(2):110–5. 10.1016/j.parkreldis.2008.03.010 18487069

[40] Wesnes KA McKeith I Edgar C: Benefits of rivastigmine on attention in dementia associated with Parkinson disease. *Neurology.* 2005;65(10):1654–6. 10.1212/01.wnl.0000184517.69816.e9 16301500

[41] Royall DR Cordes JA Polk M: CLOX: an executive clock drawing task. *J Neurol Neurosurg Psychiatry.* 1998;64(5):588–94. 10.1136/jnnp.64.5.588 9598672 PMC2170069

[42] Wechsler D: A standardized memory scale for clinical use. *J Psychol.* 1945;19(1):87–95. 10.1080/00223980.1945.9917223

[43] Wilde NJ Strauss E Tulsky DS: Memory span on the Wechsler Scales. *J Clin Exp Neuropsychol.* 2004;26(4):539–49. 10.1080/13803390490496605 15512941

[44] Matsuoka T Narumoto J Shibata K: Neural correlates of performance on the different scoring systems of the clock drawing test. *Neurosci Lett.* 2011;487(3):421–5. 10.1016/j.neulet.2010.10.069 21055445

[45] Calamia M Markon K Denburg NL: Developing a short form of Benton's Judgment of Line Orientation Test: an item response theory approach. *Clin Neuropsychol.* 2011;25(4):670–84. 10.1080/13854046.2011.564209 21469016 PMC3094715

[46] Binetti G Cappa SF Magni E: Visual and spatial perception in the early phase of Alzheimer's disease. *Neuropsychology.* 1998;12(1):29–33. 10.1037/0894-4105.12.1.29 9460732

[47] Rapport LJ Millis SR Bonello PJ: Validation of the Warrington theory of visual processing and the Visual Object and Space Perception Battery. *J Clin Exp Neuropsychol.* 1998;20(2):211–20. 10.1076/jcen.20.2.211.1169 9777475

[48] Bonello PJ Rapport LJ Millis SR: Psychometric properties of the visual object and space perception battery in normal older adults. *Clin Neuropsychol.* 1997;11(4):436–42. 10.1080/13854049708400475

[49] Herrera-Guzmán I Peña-Casanova J Lara JP: Influence of age, sex, and education on the Visual Object and Space Perception Battery (VOSP) in a healthy normal elderly population. *Clin Neuropsychol.* 2004;18(3):385–94. 10.1080/1385404049052421 15739810

[50] Lawrence AD Watkins LH Sahakian BJ: Visual object and visuospatial cognition in Huntington's disease: implications for information processing in corticostriatal circuits. *Brain.* 2000;123(Pt 7):1349–64. 10.1093/brain/123.7.1349 10869048

[51] Hazel CA Elliott DB: The dependency of logMAR visual acuity measurements on chart design and scoring rule. *Optom Vis Sci.* 2002;79(12):788–92. 10.1097/00006324-200212000-00011 12512687

[52] Hussain B Saleh GM Sivaprasad S: Changing from Snellen to LogMAR: debate or delay? *Clin Experiment Ophthalmol.* 2006;34(1):6–8. 10.1111/j.1442-9071.2006.01135.x 16451251

[53] Woods R Wood JM: The role of contrast sensitivity charts and contrast letter charts in clinical practice. *Clin Experiment Optometry.* 1995;78(2):43–57. 10.1111/j.1444-0938.1995.tb00787.x

[54] Goetz CG Tilley BC Shaftman SR: Movement Disorder Society-sponsored revision of the Unified Parkinson's Disease Rating Scale (MDS-UPDRS): scale presentation and clinimetric testing results. *Mov Disord.* 2008;23(15):2129–70. 10.1002/mds.22340 19025984

[55] Bhidayasiri R Tarsy D: Parkinson’s Disease: Hoehn and Yahr Scale.2012;4–5. 10.1007/978-1-60327-426-5_2

[56] Nieuwboer A Rochester L Herman T: Reliability of the new freezing of gait questionnaire: agreement between patients with Parkinson's disease and their carers. *Gait Posture.* 2009;30(4):459–63. 10.1016/j.gaitpost.2009.07.108 19660949

[57] Giladi N Tal J Azulay T: Validation of the freezing of gait questionnaire in patients with Parkinson's disease. *Mov Disord.* 2009;24(5):655–61. 10.1002/mds.21745 19127595

[58] Yardley L Beyer N Hauer K: Development and initial validation of the Falls Efficacy Scale-International (FES-I). *Age Ageing.* 2005;34(6):614–9. 10.1093/ageing/afi196 16267188

[59] Cohen RG Chao A Nutt JG: Freezing of gait is associated with a mismatch between motor imagery and motor execution in narrow doorways, not with failure to judge doorway passability. *Neuropsychologia.* 2011;49(14):3981–8. 10.1016/j.neuropsychologia.2011.10.014 22027173 PMC3260879

[60] Verghese J Kuslansky G Holtzer R: Walking while talking: effect of task prioritization in the elderly. *Arch Phys Med Rehabil.* 2007;88(1):50–3. 10.1016/j.apmr.2006.10.007 17207675 PMC1894901

[61] Morris ME Iansek R Matyas TA: Stride length regulation in Parkinson's disease. Normalization strategies and underlying mechanisms. *Brain.* 1996;119(Pt 2):551–68. 10.1093/brain/119.2.551 8800948

[62] Azulay JP Mesure S Amblard B: Visual control of locomotion in Parkinson's disease. *Brain.* 1999;122(Pt 1):111–20. 10.1093/brain/122.1.111 10050899

[63] Bulling A Roggen D: Recognition of Visual Memory Recall Processes Using Eye Movement Analysis. *UbiComp’11.*Beijing, China.2011;455–464. 10.1145/2030112.2030172

[64] Turner J Bulling A Gellersen H: Combining Gaze with Manual Interaction to Extend Physical Reach. *PETMEI'11.*Beijing, China.2011;33–36. 10.1145/2029956.2029966

[65] Vidal M Bulling A Gellersen H: Analysing EOG Signal Features for the Discrimination of Eye Movements with Wearable Devices. *PETMEI’11.*Beijing, China.2011;15–20. 10.1145/2029956.2029962

[66] Stuart S Galna B Lord S: Quantifying Saccades While Walking: validity of a novel velocity-based algorithm for mobile eye tracking. *Conf Proc IEEE Eng Med Biol Soc.*Chicago, Illinois, USA: IEEE.2014;2014:5739–42. 10.1109/EMBC.2014.6944931 25571299

[67] Holmqvist K Nystrom M Andersson R: Eye tracking: A comprehensive guide to methods and measures.Oxford, UK: Oxford University Press.2011 Reference Source

[68] Huang WN VanSwearingen JM Brach JS: Gait variability in older adults: observational rating validated by comparison with a computerized walkway gold standard. *Phys Ther.* 2008;88(10):1146–53. 10.2522/ptj.20070243 18719005 PMC2557053

[69] Duchowski A: Eye-Tracking-Methodology-Theory-and-Practice.London: Springer.2007 10.1007/978-1-84628-609-4

[70] Chapman GJ Hollands MA: Age-related differences in visual sampling requirements during adaptive locomotion. *Exp Brain Res.* 2010;201(3):467–78. 10.1007/s00221-009-2058-0 19882147

[71] Lohnes CA Earhart GM: Saccadic eye movements are related to turning performance in Parkinson disease. *J Parkinsons Dis.* 2011;1(1):109–18. 10.3233/JPD-2011-11019 22216083 PMC3247902

[72] Lohnes CA Earhart GM: Movement orientation switching with the eyes and lower limb in Parkinson disease. *Parkinsonism Relat Disord.* 2012;18(5):462–8. 10.1016/j.parkreldis.2012.01.002 22261609 PMC3354036

[73] Lohnes CA Earhart GM: Effect of subthalamic deep brain stimulation on turning kinematics and related saccadic eye movements in Parkinson disease. *Exp Neurol.* 2012;236(2):389–94. 10.1016/j.expneurol.2012.05.001 22580213 PMC3392464

[74] Lord S Galna B Coleman S: Cognition and gait show a selective pattern of association dominated by phenotype in incident Parkinson's disease. *Front Aging Neurosci.* 2014;6:249. 10.3389/fnagi.2014.00249 25374538 PMC4205301

[75] Moes E Lombardi KM: The relationship between contrast sensitivity, gait, and reading speed in Parkinson's disease. *Neuropsychol Dev Cogn B Aging Neuropsychol Cogn.* 2009;16(2):121–32. 10.1080/13825580802233418 18688759

[76] Wood JM Lacherez PF Black AA: Postural stability and gait among older adults with age-related maculopathy. *Invest Ophthalmol Vis Sci.* 2009;50(1):482–7. 10.1167/iovs.08-1942 18791170

[77] Anastasopoulos D Ziavra N Savvidou E: Altered eye-to-foot coordination in standing parkinsonian patients during large gaze and whole-body reorientations. *Mov Disord.* 2011;26(12):2201–11. 10.1002/mds.23798 21661049

[78] Lee HC Yanting Chee D Selander H: Is it reliable to assess visual attention of drivers affected by Parkinson's disease from the backseat?-a simulator study. *Emerg Health Threats J.* 2012;5. 10.3402/ehtj.v5i0.15343 22461850 PMC3290114

[79] Vitorio R Lirani-Silva E Barbieri FA: The role of vision in Parkinson's disease locomotion control: free walking task. *Gait Posture.* 2012;35(2):175–9. 10.1016/j.gaitpost.2011.09.002 21962407

[80] Vitorio R Lirani-Silva E Barbieri FA: Influence of visual feedback sampling on obstacle crossing behavior in people with Parkinson's disease. *Gait Posture.* 2013;38(2):330–4. 10.1016/j.gaitpost.2012.12.019 23347768

[81] Expósito-Ruiz M Pérez-Vicente S Rivas-Ruiz F: Statistical inference: hypothesis testing. *Allergol Immunopathol (Madr).* 2010;38(5):266–77. 10.1016/j.aller.2010.06.003 20817378

[82] Stuart S Lord S Hill E: Gait in Parkinson’s disease: A visuo-cognitive challenge. *Neurosci Biobehav Rev.* 2016;62:76–88. 10.1016/j.neubiorev.2016.01.002 26773722

[83] Musil CM Jones SL Warner CD: Structural equation modeling and its relationship to multiple regression and factor analysis. *Res Nurs Health.* 1998;21(3):271–281. 10.1002/(SICI)1098-240X(199806)21:3<271::AID-NUR10>3.0.CO;2-G 9609512

[84] Hayes AF: Beyond Baron and Kenny: Statistical mediation analysis in the new millennium. *Commun Monogr.* 2009;76(4): 408–420. 10.1080/03637750903310360

[85] Bentler PM Chou CP: Practical Issues in Structural Modeling. *Sociol Method Res.* 1987;16(1):78–117. 10.1177/0049124187016001004

[86] Byrne BM: Structural equation modeling with AMOS: Basic concepts, applications, and programming.2nd ed. *Multivariate Applications Series* Routledge: Taylor and Francis Group.2013 Reference Source

[87] Kline RB: Principles and Practice of Structural Equation Modeling.3rd ed, ed. T.D. Little, New York: Guildford Publishing Group,2011 Reference Source

[88] Mueller R Hancock GR: Best practices in structural equation modeling.In *Best practices in quantitative methods* J.W.E. Osborne, Editor, Sage: Thousand Oaks, CA.2008;488–508. 10.4135/9781412995627.d38

[89] Xiong B Skitmore M Xia B: A critical review of structural equation modeling applications in construction research. *Automat Constr.* 2015;49(Part A):59–70. 10.1016/j.autcon.2014.09.006

[90] Hancock GR Mueller RO: The Reliability Paradox in Assessing Structural Relations Within Covariance Structure Models. *Educ Psychol Meas.* 2011;71(2):306–324. 10.1177/0013164410384856

[91] Menz HB Lord SR Fitzpatrick RC: A structural equation model relating impaired sensorimotor function, fear of falling and gait patterns in older people. *Gait Posture.* 2007;25(2):243–9. 10.1016/j.gaitpost.2006.04.005 16697643

[92] Sacrey LA Clark CA Whishaw IQ: Music attenuates excessive visual guidance of skilled reaching in advanced but not mild Parkinson's disease. *PLoS One.* 2009;4(8):e6841. 10.1371/journal.pone.0006841 19718260 PMC2729398

[93] Sacrey LA Travis SG Whishaw IQ: Drug treatment and familiar music aids an attention shift from vision to somatosensation in Parkinson's disease on the reach-to-eat task. *Behav Brain Res.* 2011;217(2):391–8. 10.1016/j.bbr.2010.11.010 21073905

