# Supplementary material for: A protocol to examine vision and gait in Parkinson’s disease: impact of cognition and response to visual cues
Source: F1000Res. 2016 Mar 24;4:1379. Originally published 2015 Nov 30. [Version 2] doi: 10.12688/f1000research.7320.2 (PMC4821288; doi:10.12688/f1000research.7320.2)
Supplement: Peer review history

# Peer review: Referee response for version 2

**DOI:** 10.5256/f1000research.8311.r13070

**Published:** 2016-04-04

**Authors:** Rebecca J Reed-Jones

**Affiliations:** 1 Department of Applied Human Sciences, University of Prince Edward Island, Charlottetown, PE, Canada

The authors have addressed the suggestions made by the reviewers well. The methods are clearly laid out. In addition, the section describing how each analysis addresses a particular aim in the study is nicely written. I believe the protocol as outlined will provide interesting data that will contribute significantly to current literature regarding vision and cognition during gait in individuals with Parkinson's disease.

I have read this submission. I believe that I have an appropriate level of expertise to confirm that it is of an acceptable scientific standard.

# Peer review: Referee response for version 1

**DOI:** 10.5256/f1000research.7889.r11399

**Published:** 2015-12-10

**Authors:** Rodrigo Vitório

**Affiliations:** 1 Sun Life Financial Movement Disorders Research & Rehabilitation Centre, Wilfrid Laurier University, Waterloo, ON, Canada

In this paper, S. Stuart and colleagues present an elegant and thorough protocol for testing visuo-cognition during gait. Authors aim to examine the contribution of cognition and vision in gait in Parkinson's Disease (independent roles and interaction between both functions), and the role of visuo-cognition in gait in Parkinson's Disease.

The novelty of current study is taking into consideration the combined and interactive impact that cognitive and visual function impairments have on gait in Parkinson's Disease. To date, previous studies in this field have investigated the independent contribution of cognition and vision to gait in Parkinson's Disease.

The described experimental design, including controls and methods, is totally adequate. Although the current version does not report the results, my expectation is that this study, once complete, will impact the field.

I have read this submission. I believe that I have an appropriate level of expertise to confirm that it is of an acceptable scientific standard.

# Response

**Published:** 2016-03-16

**Authors:** Sam Stuart

**Affiliations:** Newcastle University, UK

We thank the reviewer for their time and comments.

# Peer review: Referee response for version 1

**DOI:** 10.5256/f1000research.7889.r11401

**Published:** 2015-12-07

**Authors:** Rebecca J Reed-Jones

**Affiliations:** 1 Department of Applied Human Sciences, University of Prince Edward Island, Charlottetown, PE, Canada

This protocol seeks to understand whether visual-cognition influences gait performance and visual sampling behaviour in individuals with Parkinson's disease. Overall the protocol is well written and the details provided are sufficient to replicate the study. The sample sizes are good for gait and eye tracking data analysis. The tests used for evaluation of cognition and visual spatial ability are validated and appropriate tests. However, there are several methods and/or analyses the authors should consider in their protocol that will help to address their aims. In addition, there are several minor additions that the authors should also consider.

Major points:

1. Is the 4-point calibration matrix for the eye tracker sufficient? The accuracy of eye trackers  increases with the number of points used in the calibration. Some indication as to the accuracy of using only four points should be made. In addition, what was the size of the calibration field used? The calibration field should be as large as the filed of view intended for the activity. In gait, the visual field is relatively large and therefore making the calibration field as large as possible is important.
2. For visual sampling dependent variables, I would recommend the addition of examining areas of interest. In addition to whether participants are moving their eyes (how much, how often and how quickly), WHAT they are looking at is also of value. This is particularly relevant to the research question of understanding the influence of cognition as WHAT they are looking at may give you an indication of attention to particular objects or areas that may or may not be relevant to the task. For example, some work done by Shirley Rietdyk that was presented at ISPGR in Seville showed some interesting results of increased obstacle collisions when young adults looked off the travel pathway.
3. Given the number of visual spatial and cognitive tests used to assess these domains, would a more sophisticated regression model be more valuable? The use of principal component analyses (PCAs) or separate  hierarchical  linear  regression  models  could examine  the  interrelationships  of  the  variables. Details on these types of analyses that may be useful can be found in the following papers.
  Pua  YH,  Liang  Z,  Ong  PH,  Bryant  AL,  Lo  NN,  Clark  RA. (2011)  Associations  of  knee extensor  strength  and  standing  balance  with  physical  function  in  knee  osteoarthritis.  Arthritis  Care  and  Research  2011;63(12):1706–14. doi: 10.1002/acr.20615 ^1^
  Reed-Jones  RJ,  et  al.  WiiFit ^TM^ Plus  balance  test  scores  for  the  assessment  of  balance  and  mobility  in older  adults. Gait & Posture 36(3):430-3. doi:10.1016/j.gaitpost.2012.03.027 ^2^

Minor points:

1. The aims are stated clearly, however when it comes to the methods there are a large number of tests described. Reading through the tests in the methods it is not clear what these will be used for and how they address the aims of the research. Perhaps within the aims section an additional sentence or two that indicates how each aim will be tested would help the reader to follow along with the tests described in the methods more clearly.
2. The authors provide details on the inclusion/exclusion criteria for participants. However, for the PD participants, a summary of the demographics of the participants in the study should be provided. In particular, the distribution of H&Y stages in the group, the distribution of medication versus DBS, versus a combination of the two.

I have read this submission. I believe that I have an appropriate level of expertise to confirm that it is of an acceptable scientific standard, however I have significant reservations, as outlined above.

## References

[1] PuaYHLiangZOngPHBryantALLoNNClarkRA: Associations of knee extensor strength and standing balance with physical function in knee osteoarthritis. *Arthritis Care Res (Hoboken)*.2011;63(12) : 10.1002/acr.20615 1706-14 10.1002/acr.20615 21905251

[2] Reed-JonesRJDorgoSHitchingsMKBaderJO: WiiFit™ Plus balance test scores for the assessment of balance and mobility in older adults. *Gait Posture*.2012;36(3) : 10.1016/j.gaitpost.2012.03.027 430-3 10.1016/j.gaitpost.2012.03.027 22534562PMC3407275

# Response

**Published:** 2016-03-16

**Authors:** Sam Stuart

**Affiliations:** Newcastle University, UK

We thank the reviewer for their time and comments which we believe have improved the manuscript. Please find below a list of reviewer comments and our reply.

Comments (Q) & Response (A)

Major Points:

*Q: Is the 4-point calibration matrix for the eye tracker sufficient? The accuracy of eye trackers increases with the number of points used in the calibration. Some indication as to the accuracy of using only four points should be made. In addition, what was the size of the calibration field used? The calibration field should be as large as the field of view intended for the activity. In gait, the visual field is relatively large and therefore making the calibration field as large as possible is important.*

A: The four-point calibration procedure has been developed by the manufacturer and we are unable to increase or decrease the number of points used. We agree that it may increase the accuracy of the calibration with more points, but this has yet to be investigated. We calibrated the eye tracker to the dimensions of the gaitlab where the individuals would walk, which meant that the calibration was as large as possible. This was done by placing four cones with markers on top in the area to be walked through, which were used as the four points for the calibration.

*Q: For visual sampling dependent variables, I would recommend the addition of examining areas of interest. In addition to whether participants are moving their eyes (how much, how often and how quickly), WHAT they are looking at is also of value. This is particularly relevant to the research question of understanding the influence of cognition as WHAT they are looking at may give you an indication of attention to particular objects or areas that may or may not be relevant to the task. For example, some work done by Shirley Rietdyk that was presented at ISPGR in Seville showed some interesting results of increased obstacle collisions when young adults looked off the travel pathway.*

A: Examining areas of interest is certainly a topic for future investigation in this field of research. However mobile eye-tracking technology is not capable of automatically performing area of interest analysis with sufficient accuracy for our purpose. Currently only frame-by-frame manual analysis of the area of interest data is possible, which with large numbers of participants and trials can be time consuming.

Our recent mobile eye-tracker accuracy and reliability article ( Stuart *et al.*, 2016a) also showed that during walking the device (i.e. the cross hair on the field camera, that signifies where an individual is looking) can be up to 8 degrees off target, which can be the difference between looking at an obstacle and not looking at one. This does not affect the temporal data, as eye movements are still detected regardless of location of the cross hair on the field camera, but may impact area of interest analysis. Therefore the current study will focus on the temporal data, specifically saccade frequency during gait.

*Q: Given the number of visual spatial and cognitive tests used to assess these domains, would a more sophisticated regression model be more valuable? The use of principal component analyses (PCAs) or separate  hierarchical  linear  regression  models  could examine  the  interrelationships  of  the  variables. Details on these types of analyses that may be useful can be found in the following papers.*

*Pua  YH,  Liang  Z,  Ong  PH,  Bryant  AL,  Lo  NN,  Clark  RA. (2011)  Associations  of  knee extensor  strength  and  standing  balance  with  physical  function  in  knee  osteoarthritis.  Arthritis  Care  and  Research  2011;63(12):1706–14. doi: 10.1002/acr.20615 ^1^*

*Reed-Jones  RJ,  et  al.  WiiFit ^TM^ Plus  balance  test  scores  for  the  assessment  of  balance  and  mobility  in older  adults. Gait & Posture 36(3):430-3. doi:10.1016/j.gaitpost.2012.03.027 ^2^*

A: The contribution of cognitive and visual functions to visual sampling will be investigated based upon our *a priori* hypothesis that cognitive and visual functions, as well as demographic features will be associated with visual sampling (specifically saccade frequency).

This will be done using multiple linear regression, performed in four separate steps;

Step 1: Demographics (Age, UPDRS III, MoCA, GDS-15)

Step 2: Cognition (Attention, Executive function, Visuo-spatial ability, Working memory)

Step 3: Visual Functions (visual acuity, contrast sensitivity)

Step 4: Visuo-cognition (combination of all of the variables in the above steps)

Variables entered into the steps will be determined through univariate and bivariate analysis. Variables that best represent each cognitive outcome will be used within the analysis.Text has been added to the statistical analysis section which describes this analysis.

Structural equation modelling (SEM) will be used to examine our *a priori* model of visuo-cognition in gait in PD (For a review pertaining to the model see; ( Stuart *et al.*, 2016b). This model will examine the inter-relationships between cognition, visual function, visual sampling (saccade frequency) and gait in PD. This technique is an ideal statistical method for assessing *a priori* hypotheses, as it allows for hypothesised interactions to be represented within the model. SEM also provides direct and indirect relationships between variables, which are not provided by regression analysis. This has been added to the statistical analysis section of the article.

Minor points:

*Q: The aims are stated clearly, however when it comes to the methods there are a large number of tests described. Reading through the tests in the methods it is not clear what these will be used for and how they address the aims of the research. Perhaps within the aims section an additional sentence or two that indicates how each aim will be tested would help the reader to follow along with the tests described in the methods more clearly.*

**

A: The study aims with specific analysis are now provided within the statistical analysis section of the article.

*Q: The authors provide details on the inclusion/exclusion criteria for participants. However, for the PD participants, a summary of the demographics of the participants in the study should be provided. In particular, the distribution of H&Y stages in the group, the distribution of medication versus DBS, versus a combination of the two.*

A: A brief summary table for all participant demographic and clinical features is now provided within the article; Table 3. There are no individuals within the study that had Deep Brain Stimulation (DBS).
